# Antecedent acute cycling exercise affects attention control: an ERP study using attention network test

**DOI:** 10.3389/fnhum.2015.00156

**Published:** 2015-04-09

**Authors:** Yu-Kai Chang, Caterina Pesce, Yi-Te Chiang, Cheng-Yuh Kuo, Dong-Yang Fong

**Affiliations:** ^1^Graduate Institute of Athletics and Coaching Science, National Taiwan Sport UniversityTaoyuan City, Taiwan; ^2^Department of Movement, Human and Health Sciences, Italian University Sport and Movement “Foro Italico”Rome, Italy; ^3^Physical Education Office, National Taipei University of TechnologyTaipei, Taiwan

**Keywords:** alerting, orienting, executive function, interference control, spinning

## Abstract

The purpose of this study was to investigate the after-effects of an acute bout of moderate intensity aerobic cycling exercise on neuroelectric and behavioral indices of efficiency of three attentional networks: alerting, orienting, and executive (conflict) control. Thirty young, highly fit amateur basketball players performed a multifunctional attentional reaction time task, the attention network test (ANT), with a two-group randomized experimental design after an acute bout of moderate intensity spinning wheel exercise or without antecedent exercise. The ANT combined warning signals prior to targets, spatial cueing of potential target locations and target stimuli surrounded by congruent or incongruent flankers, which were provided to assess three attentional networks. Event-related brain potentials and task performance were measured during the ANT. Exercise resulted in a larger P3 amplitude in the alerting and executive control subtasks across frontal, central and parietal midline sites that was paralleled by an enhanced reaction speed only on trials with incongruent flankers of the executive control network. The P3 latency and response accuracy were not affected by exercise. These findings suggest that after spinning, more resources are allocated to task-relevant stimuli in tasks that rely on the alerting and executive control networks. However, the improvement in performance was observed in only the executively challenging conflict condition, suggesting that whether the brain resources that are rendered available immediately after acute exercise translate into better attention performance depends on the cognitive task complexity.

## Introduction

Growing evidence is converging to suggest that various cognitive functions and functional modalities of the brain are improved by physical exercise. The conclusions drawn by meta-analytic reviews indicate that single bouts of exercise have an overall small positive effect on cognitive performance (Lambourne and Tomporowski, 2010; Chang et al., 2012). However, effect size largely depends on moderators that act on the exercise-cognition relation, such as the physical and cognitive task characteristics, time relation between physical and cognitive task performance, and individual characteristics of the performers.

The substantial progress that this area of research has experienced in the previous decades is characterized by linked theoretical and methodological advancements. The development of the theoretical underpinnings, which are centered on the search for the mechanisms responsible for the exercise-cognition interaction, has moved from an exercise-arousal-cognitive performance interaction model to interdisciplinary rationales, which are methodologically supported by the growing neuroscience perspective on the neural correlates of the effects of exercise on cognition (McMorris, 2008).

The joint analysis of behavioral and neuroelectric measures with high temporal resolution (event-related brain potentials, ERPs) facilitates the identification of the different pathways through which the neural modulation by physical exercise translates into more, less or equally efficient cognitive performance (Pontifex and Hillman, 2008; Hillman et al., 2009). The majority of ERP studies of the acute exercise-cognition interaction in adulthood and childhood have primarily focused on the P3 component of ERPs. P3 is a positive-going component observed in the stimulus-locked ERP waveform. Its amplitude and latency reflect the amount of attentional resources allocated to external events in the environment and the cognitive processing speed, respectively (Hillman et al., 2011). The main result in physical exercise research is an exercise-induced increase in P3 amplitude that is behaviorally paralleled by enhanced cognitive efficiency after exercise cessation (Hillman et al., 2003; Kamijo et al., 2004, 2007). This finding suggests that the cognitive benefits associated with acute exercise may be mediated by a short-term enhancement of mental resources that can be allocated to a subsequent cognitive task.

These ERP studies employed tasks (e.g., Eriksen flanker and go-nogo tasks) that reflect the progressive focusing of researchers’ interests toward exercise effects on higher-level cognitive functions from both behavioral and neurophysiological perspectives (Etnier and Chang, 2009). Nevertheless, there are aspects of cognitive functioning as attentional processes that remain underrepresented in the panorama of acute exercise and cognition research, which is surprising because the relevance of attention in sports and particularly in strategic open-skill sports is undoubtedly recognized (Memmert, 2009). Given the predominant role of visual information selection for action in many sports, the few existing studies of the exercise-attention association in adolescent, young adult and old adult athletes and physically active individuals have primarily focused on visual attention (Pesce et al., 2007a,b, 2011; Cereatti et al., 2009; Huertas et al., 2011; Hüttermann and Memmert, 2014).

Attention is, however, a complex construct that encompasses multiple functionally and anatomically segregated but interacting networks that according to the authoritative attention network theory by Posner and Petersen (1990), carry out the functions of alerting, orienting and executive (or conflict) control. The alerting network, which comprises right frontal and parietal cortex and thalamus, is related to arousal and vigilance and ensures sensitivity to incoming stimuli and readiness to react. The orienting network, which includes parietal cortex and frontal eye fields, enables the selection of information by disengaging, shifting and reengaging attention in the visual space. The executive control network is also referred to as the conflict network because it is involved in self-regulation and conflict resolution between thoughts, emotions and overt responses. Its substrate includes the anterior cingulate and lateral ventral prefrontal cortex (Fan et al., 2002; Posner and Rothbart, 2007).

The efficiency of these networks has been studied by combining in one task, the ANT (Posner and Petersen, 1990), warning signals prior to targets (alerting), cues that direct attention toward potential target locations (orienting) and target stimuli surrounded by congruent or incongruent flankers (executive control). The ANT also allows studying how these attentional networks interact. While the ANT has been shown to be a useful tool in several applied and clinical settings, to our knowledge, it has only been applied once in a modified version (attention network test-interactions, ANT-I; Callejas et al., 2004) to the study of physical exercise effects (Huertas et al., 2011). The authors tested two intensity levels of concomitant exercise (80 and 95% of the individual workload at lactate threshold) and found that while intense exercise enhanced general reaction speed, moderate exercise modulated the alerting function, as indicated by a reduction in the alerting effect. Specifically, an exercise-induced increment in tonic alertness seemed to facilitate RT performance in absence of warning signals. Instead, neither exercise intensity levels altered the functioning of the orienting and executive control networks.

Apart from this study, only piecemeal and inconsistent evidence of acute exercise effects on the functioning of the three attentional networks is available from studies that differ in exercise timing and intensity. Phasic and tonic alertness appear to be affected by intense exercise in opposite directions, with a detrimental effect on response to phasic alerting stimuli (Mahoney et al., 2007), but a beneficial effect on tonic alertness (Smit et al., 2005). Furthermore, orienting appears to be differentially affected by moderate to intense exercise depending on whether attention is cued to orient automatically (Sanabria et al., 2011; Llorens et al., 2014) or intentionally with a higher or lesser degree of executive control (Pesce et al., 2007a,b, 2011; Cereatti et al., 2009). Notably, the majority of the cited studies on alerting and orienting are in-task exercise studies, while only Llorens et al. (2014) evaluated the after-effects of acute exercise (off-task) and Sanabria et al. (2011) joined in-task and off-task measurements. Instead, acute exercise effects on conflict control have mainly been studied with off-task exercise and demonstrated either a general reaction time (RT) facilitation after exercise under both congruent and incongruent conditions (Kamijo et al., 2007) or no exercise after-effects (Hillman et al., 2003). A thorough comparison is also limited by different and differently measured exercise intensity: 130–150 heart rate (HR) range (Smit et al., 2005), HR corresponding to the individual anaerobic threshold (Llorens et al., 2014) or 85% of it (Sanabria et al., 2011), or 60% HR reserve (Pesce et al., 2007a,b, 2011; Cereatti et al., 2009), or target HR corresponding to light, moderate, or hard (Kamijo et al., 2007), or moderate-to-hard (Hillman et al., 2003) perceived exertion.

In sum, there is a paucity of acute exercise studies examining the three attentional networks in combination (Huertas et al., 2011) and ERP studies of the exercise-attention relation almost exclusively focus on the conflict control network only. The present study examined whether an acute bout of moderate intensity exercise influences the functioning of the attentional networks from both behavioral and neuroscientific perspective. The exercise bout chosen for the present study was spinning. It shares characteristics of static cycling with exercise bouts commonly employed in acute exercise and cognition studies, but embedded in an ecological form of indoor group exercise that has gained popularity within recent years in the fitness world to achieve the recommended levels of physical activity (Lopez-Minarro and Muyor Rodríguez, 2010).

Thus, the general purpose of the study was to examine the after-effects of an acute bout of exercise commonly used in fitness training on the attention networks and provide insight into the neural mechanisms underlying attentional performance. From a behavioral perspective, the specific aims of the study were to verify whether (1) the effect of acute in-task exercise on the alerting network performance found by Huertas et al. (2011) is also present after exercise cessation and (2) there are beneficial effects for the orienting and conflict control networks that might have been outweighed by dual task effects in Huertas et al. (2011) study. From a neuroscientific perspective, we expected that the moderate intensity of the cycling exercise bout would lead to increased allocation of neuroelectric resources, as reflected in larger P3 amplitude that has been consistently found after exercise in other tasks involving executive control (Hillman et al., 2003; Kamijo et al., 2004, 2007). Regarding P3 latency, we aimed at understanding whether after moderate exercise, the expected general RT facilitation and the specific reduction of attentional effects on tasks relying on orienting and executive control networks (Pesce et al., 2007a,b) may be linked to enhanced stimulus evaluation and cognitive processing speed, as reflected in shorter P3 latency. Indeed, in previous ERP studies, P3 latency seemed more sensitive to acute exercise under attention demanding conditions (Hillman et al., 2003; Kamijo et al., 2007).

To isolate the effects of exercise on the different components of attentional performance, we controlled for individual and task constraints that potentially act on the exercise–attention relationship (Pesce, 2009). Because the physical exercise intensity and the individual fitness level may interactively moderate the relationship of interest, we chose a moderate exercise intensity and selected participants with a high fitness level, which enables larger benefits to be obtained from acute exercise compared with a lower fitness level (Chang et al., 2014). Additionally, the coordinative complexity of the movement task and the individual motor proficiency level may jointly affect the exercise-attention relationship. Therefore, we reduced the constraints on spinning performance by allowing the participants to freely choose the pedaling rate, thus reducing the need to allocate cognitive resources to the physical movements inherent in exercise and the risk of attentional fatigue (Davranche and Audiffren, 2004). We also trained the participants to a sufficient level of proficiency in coordinating spinning movements. Finally, the size and selectivity of acute exercise effects on cognition also appear to depend on the interplay between cognitive task complexity and individual cognitive expertise (Pesce, 2009). Therefore, we selected participants who practiced a strategic team sport that requires cognitive expertise and used a multifunctional attentional task, the ANT, which enables the manipulation of cognitive task complexity and the isolation of different components of attentional efficiency.

## Materials and Methods

### Participants

The participants were 30 young, highly fit college students recruited from an amateur basketball team of the National Taipei University of Technology, Taiwan. The participants practiced regular exercise and basketball training of moderate intensity twice per week for 2 h per session. In addition, the participants had experienced a group spinning exercise course for 8 weeks once per week.

Eligible participants included individuals who met the criteria of right-hand dominance, normal or corrected-to-normal vision, absence of intellectual disability, no reported history of neurological or psychiatric illness or other pathological conditions that could potentially influence study outcomes, and a passing Physical Activity Readiness Questionnaire (PAR-Q) score to ensure participant safety when performing a fitness test and an acute exercise bout according to published guidelines (American College of Sports Medicine, 2013).

The participants were randomly assigned to the exercise or control groups. All participants read and signed a written informed consent form prior to participation in the study. The protocol was reviewed and approved by the National Taiwan Sport University Institutional Review Board and the experiment conformed to the relevant regulatory standards. **Table 1** presents the participants’ demographic characteristics.

**Table 1:** Summary of the participants’ demographic and exercise characteristics.

Variables	Exercise group	Control group
*n*	15	15
Gender F/M	7/8	7/8
Age (year)	21.67 ± 3.77	20.17 ± 1.53
Height (cm)	169.60 ± 8.07	170.87 ± 7.81
Weight (kg)	63.13 ± 7.32	62.27 ± 7.16
BMI (kg.m^-2^)	21.91 ± 1.60	21.32 ± 1.99
Education (years)	16.07 ± 1.10	15.67 ± 0.82
VO_2max_ (men)	53.69 ± 1.71	52.50 ± 2.60
VO_2max_ (women)	42.53 ± 1.21	42.50 ± 1.69
IPAQ (MET)	4929.34 ± 1408.94	4934.13 ± 2561.63
Resting HR	69.87 ± 3.29	69.60 ± 2.56
Treatment HR	–	144.03 ± 1.99
RPE	–	14.80 ± 1.82

*BMI, body mass index; IPAQ, International Physical Activity Questionnaire (Metabolic equivalents/week); Resting HR, heart rate assessed at resting status; Treatment HR, heart rate assessed during the treatment; RPE, Rating of Perceived Exertion.*

### Attention Network Test

**FIGURE 1:**
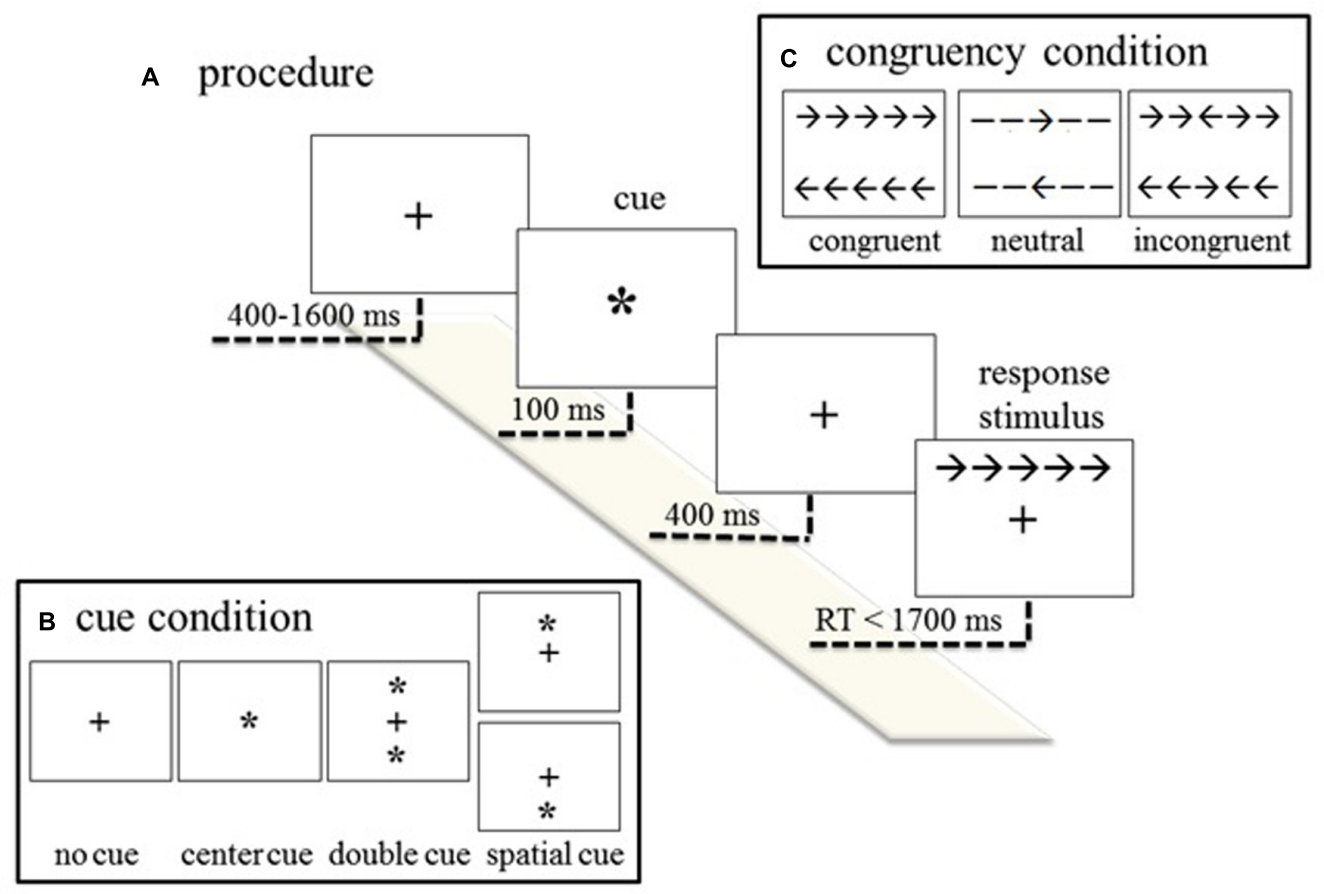
**Illustration of the experimental procedure of the ANT. (A)** An example of the sequence of events of the experimental procedure; **(B)** four cue conditions; and **(C)** response-stimulus (flanking) condition.

The computerized version of the ANT applied in the present study was modified based on Neuhaus et al. (2010) and created via Neuroscan Stim^2^ software 2.0 (Neurosoft labs, Inc., Sterling, VA, USA). Stimuli were presented on a 17-inch computer monitor with a white background located in front of the participant at a distance of 100 cm. The experimental procedure of the ANT and the timing of the event sequence within a trial are graphically represented in **Figure 1**.

A fixation cross of 0.5 cm × 0.5 cm (0.5^∘^ of visual angle) centered on the screen was presented and followed, after an interval of 400, 800, 1200, or 1600 ms in random order, by a cue that remained on the screen for 100 ms. The cueing conditions included no cue (i.e., fixation cross only), center cue (i.e., a “^*^” symbol of 0.5 cm × 0.5 cm located at the center of the screen), double cue (i.e., two “^*^” symbols of 0.5 cm × 0.5 cm each located above and below the fixation cross along the vertical meridian, 3.44^∘^ of visual angle apart center to center), and spatial cue (i.e., a “^*^” symbol located either above or below the fixation cross).

A response stimulus that consisted of five horizontally arranged elements 1.0 cm in length (a central target stimulus and four flankers) was presented 4.01^∘^ above or below the fixation cross 500 ms after cue onset (stimulus-onset-asynchrony, SOA). The flanking conditions could be neutral (i.e., a central arrow that pointed to the right or left surrounded by four lines), congruent (i.e., five arrows that all pointed in the same direction), or incongruent (i.e., five arrows with the central arrow pointed in the direction opposite to that of the flankers). The frequency of the cue and flanking conditions and their combinations were equal, and the trials were presented in a random order. The spatial cue location always validly predicted that of the response stimulus. The current ANT consisted of six blocks of 48 trials, each of which resulted in a total of 288 trials. The entire test lasted about 20–25 min.

During the ANT, the participant was instructed to press the right or left button on the designed response pad using his/her right thumb according to the direction of the central target arrow of the response stimulus as quickly and accurately as possible. The participant’s response cleared the screen for the next trial to begin; otherwise, the response stimulus remained on the screen for a maximum of 1700 ms. According to Neuhaus et al. (2010), responses shorter than 200 ms or longer than 1700 ms were considered anticipations and delayed responses, respectively, and discarded.

Three attention network effects were operationalized as the mean RT differences between specific task conditions (Fan et al., 2002): (1) alerting network effects were calculated as the RT difference between “no cue” and “double cue”; (2) orienting network effects were calculated as the RT difference between “center cue” and “spatial cue”; and (3) executive control network effects (conflict control) were calculated as the RT difference between “incongruent” and “congruent” conditions.

### EEG Acquisition and ERP Computation

The electroencephalogram (EEG) was recorded during the ANT task performance using a 64-channel EEG cap with Ag/AgCl electrodes mounted on the participant’s scalp according to the 10–10 International System (Neuroscan Quick-cap, Neuroscan Inc., Sterling, VA, USA). The 64 sensors recorded EEG activity at eight midline sites (FPz, Fz, FCz, Cz, CPz, Pz, POz, and Oz) and at 28 sites over each hemisphere (FP1/FP2, AF3/AF4, F1/F2, F3/F4, F5/F6, F7/F8, FC1/FC2, FC3/FC4, FC5/FC6, FT7/FT8, C1/C2, C3/C4, C5/C6, T7/T8, CP1/CP2, CP3/CP4, CP5/CP6, TP7/TP8, P1/P2, P3/P4, P5/P6, P7/P8, P9/P10, PO3/PO4, PO5/PO6, PO7/PO8, O1/O2, and CB1/CB2) referenced to the averaged mastoids (M1–M2). A single ground electrode was located on Fpz.

Eye movements were monitored by vertical and horizontal electro-oculogram with bipolar recoding electrodes placed at the right and left orbital canthi and at the supra- and infra-orbital of the left eye, respectively. The impedance level of all electrodes was maintained below 5 KΩ. The EEG was digitized at 1000 Hz, amplified with a Neuroscan Synamps2 amplifier (Scan 4.5, Neurosoft Labs, Inc.) with a 0.1–100 Hz band-pass, including a 60 Hz notch filter, and stored for off-line averaging.

The EEG data were then analyzed oﬄine, re-referenced to the averaged mastoids and segmented into epochs of 1100 ms, including a 100 ms pre-stimulus period for baseline correction. Epochs that exceeded ±80 μV amplitude, identified through visual inspection, were rejected as artifacts. The epochs for correct trials were sorted and averaged to obtain stimulus-locked ERPs for each experimental condition. Group-averaged ERPs were then band-pass filtered (0.1–30 Hz) to further reduce high- and low-frequency noise, particularly derived from eye movements and blinks.

The P3 of the stimulus-locked ERP component was defined as the largest positive-going peak within 300–500 ms after target stimulus onset. The P3 peak amplitude (measured with respect to the 100 ms pre-stimulus baseline) and latency were calculated at three midline electrode sites, Fz, Cz, and Pz, for subsequent statistical analysis. The selection of the midline electrodes for data analysis was based on the greatest activity for the P3 component at the group level. The grand-average ERP waveforms from all recorded electrode sites were examined to create a topographic map.

### Experimental Procedure

Each participant was tested individually in four experimental sessions within 1 day. The total experimental procedure lasted approximately 2 h. On the day before testing, the participant was instructed to avoid intake of caffeine-related beverages and physical exercise of moderate or vigorous intensity.

In Session One, the participant was asked to provide demographic information and complete a written informed consent form, as well as questionnaires that concerned health status (Health Screening Questionnaire, HSQ), exercise readiness (PAR-Q), and physical activity level (International Physical Activity Questionnaire, IPAQ; Bauman et al., 2009). The participants who met the inclusion criteria were then randomly assigned to the exercise or control groups.

In Session Two, each participant wore a HR monitor (Polar HR monitor, Mode S 610i, Finland) and remained quietly seated on a chair for 15 min for the identification of his/her resting HR. The electrode cap and other equipment needed for EEG recording was subsequently mounted and the experiment was initiated in a sound-attenuated, dimly lit room. The participant was seated comfortably in an armchair with the arms relaxed positioned palm down on the push button board so that the fingers could move freely. The participant was instructed to perform one to three practice blocks of 24 trials each until the criterion of less than a 5% error rate was met.

In Session Three, the participants who belonged to the exercise group performed a spinning wheel exercise for 40 min, including 5 min warm-up, 30 min during which they were asked to stay within a predetermined target HR zone, and 5 min of cool down. The target HR zone corresponded to 70–85% of the individual maximal HR [estimated as (220 – age), American College of Sports Medicine, 2013]. Spinning exercise in ecological training contexts is a variable-intensity exercise routine performed to music that typically requires an individual to maintain cycling speed and adjust the pedal spin resistance. Instead, for the purpose of the present study, the only exercise criterion required was that exercise intensity did not deviate from the target HR zone. Thus, the participants could freely manipulate the pedal spin resistance and speed (e.g., increase the speed while reducing the resistance or vice versa) and body position (e.g., seating or standing hill climbing position) on the spinning wheel bicycle (Keiser Inc., Fresno, CA, USA). A freely chosen pedaling rate most likely requires lower demands on attentional monitoring of the cycling movements. When exercise and cognitive testing are simultaneous, this dampens negative dual task affects (Davranche and Audiffren, 2004). When exercise is antecedent to attentional testing, as in our case, no dual task effects come into play. However, the absence of constraints on the spinning movements may reduce the risk of attentional fatigue that potentially emerges when individuals are forced to maintain a required, externally paced pedaling rate.

Heart rate and rating of perceived exertion (RPE) were recorded at 2-min intervals during spinning to obtain objective and subjective estimations of exercise intensity. The RPE range in the exercise group was only computed to ensure that the perceived workload was consistent with the HR parameter. Within the 10 min following the cessation of the spinning exercise bout, the participant started the true experimental trials of the ANT. In contrast, the participants who belonged to the control group were asked to read an exercise-related book for a period of time similar to the duration of the spinning session of the exercise group and then performed the ANT. The participants were tested in a sound-attenuated, dimly lit room at a temperature between 21 and 23^∘^C.

In Session Four, the participants performed a 1.5-mile field run test to estimate their cardiovascular fitness (VO_2max_), as indicated in the ACSM guidelines (2013, p. 88 and 91 for males and females, respectively). Session four began 10 min after the end of Session three. The participants were briefly informed regarding the purpose of the study and received approximately $15 USD for compensation.

### Statistical Analysis

The present study used a post-test only randomized experimental design. For participant characteristics and exercise manipulation, *t*-tests and χ^2^-tests were applied where appropriate to examine the mean differences in the demographic characteristics between the exercise and control groups. The analyzed variables included: gender, age, height, weight, body mass index, physical activity level, and VO_2max_. Additionally, *t*-tests were conducted on the HR data of the exercise and control groups to evaluate whether the exercise manipulation ensured that individuals in the exercise group reached at least moderate exercise intensity.

For behavioral data, to test the efficiency of the ANT in the present sample, the basic ANT effects on behavioral indices were analyzed in a 4 × 3 repeated-measures ANOVA that was separately run on the mean RTs and error rates with cue conditions (no cue, center cue, double cue, or spatial cue) and flanking conditions (congruent, neutral, or incongruent) as factors.

Additional 2 × 2 mixed-model ANOVAs were performed to test the acute exercise effect on the three attention networks: alerting, orienting, and executive control. In all three models, the between-participants factor was group (exercise versus control) and the within-participants factors were the cue condition for the alerting network (no cue versus double cue), the orienting network (center cue versus spatial cue), and the flanking condition (congruent versus incongruent) for the executive control network.

Additionally, a separated *t*-test was utilized to examine the acute exercise effects on the three attentional networks (network: alerting, orienting, and conflicting). For this aim, the following RT-differences were analyzed:

Alerting = RTno cue – RTdouble cue;

Orienting = RTcenter cue – RTspatial cue;

Executive Control = RTincongruent – RTcongruent.

For neuroelectric data, a mixed-model three-way ANOVA was conducted on the P3 peak amplitude and latency data. The factors were the same as in the RT data analysis, with the addition of the electrode site (Fz, Cz, versus Pz) as a further factor that led to a 2 (groups) × 2 (cue or flanker conditions) × 3 (electrode sites) ANOVA model.

In all analyses, the significance level was set at 0.05. In cases of significant interactions (RT and ERP data) or main effects for electrode site (ERP data), *post hoc* analyses were performed by means of pairwise comparisons (*t*-tests). Bonferroni correction of the alpha level was applied where appropriate to eliminate the problem of an inflated Type I error as a result of multiple comparisons. The partial eta square was reported as the effect size for main and interaction effects.

## Results

### Participant Characteristics and Exercise Manipulation

No differences were observed between the exercise and control groups regarding age, height, weight, body mass index, education [*t*s(28)’ < 4.37, *p*s’ > 0.13], or gender (χ2 = 0.13, *p* = 0.72). Additionally, no differences were observed between the two groups in terms of physical activity levels or cardiovascular fitness (IPAQ and estimated VO_2max_: *t*s’ < -2.7, *p*s’ > 0.79), suggesting that the participants’ demographic characteristics were similar and that the randomized post-test design was appropriate.

A significant HR difference was observed between resting HR and treatment HR for the exercise group (*t* = 74.65, *p* < 0.000); the mean HR for the exercise group was 144.03 ± 1.99, representing 73% maximal HR. Along with RPE, which ranged from 13 to 17 (mean = 14.8 ± 1.82), these findings indicate the appropriateness of this moderate intensity exercise manipulation (**Table 1**).

### Behavioral Data

#### Efficiency of the Three Attentional Networks

The 4 × 3 ANOVA revealed a significant main effect for cue condition [*F*(3,87) = 336.82, *p* < 0.001, partial η^2^ = 0.93]; no cue exhibited the longest RT (612.02 ± 11.42 ms), followed by center and double cues (570.13 ± 11.30 and 571.36 ± 11.12 ms), and spatial cue exhibited the shortest RT (494.69 ± 10.97 ms). A main effect for the flanking condition was observed [*F*(2,58) = 443.80, *p* < 0.001, partial η^2^ = 0.95]; the neutral condition exhibited the shortest RT (520.01 ± 10.57 ms), followed by the congruent condition (536.80 ± 11.21 ms), and the incongruent condition had the longest RT (629.34 ± 11.78 ms). An interaction between cue and flanking condition was also observed [*F*(6,174) = 3.98, *p* < 0.003, partial η^2^ = 0.14].

**FIGURE 2:**
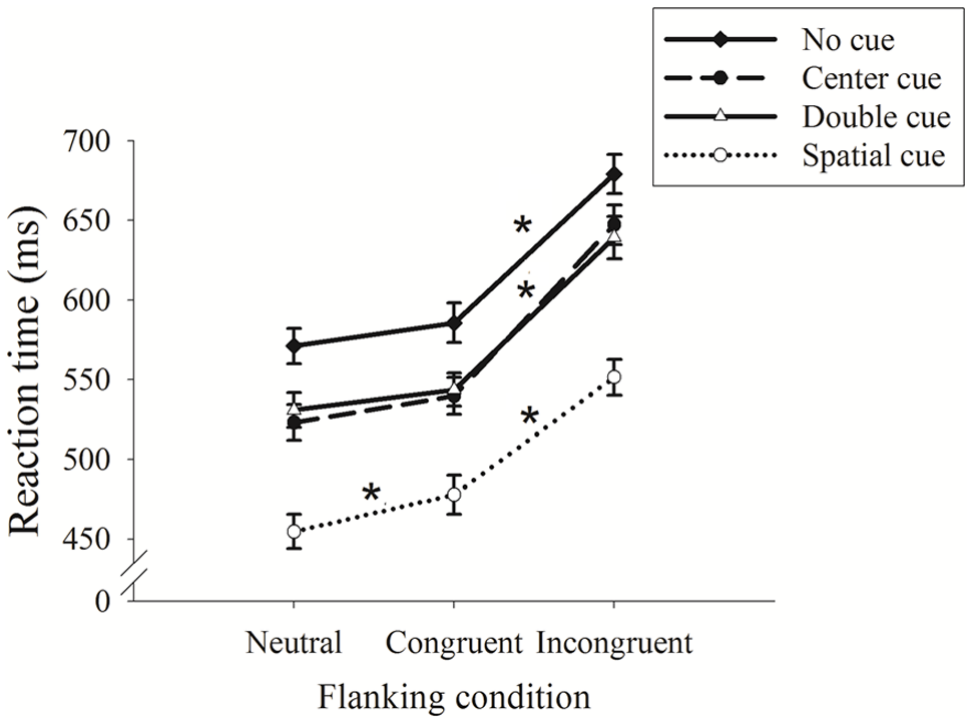
**Reaction time (RT) as a function of the cue and flanking conditions (mean ± SE)**. ^∗^*p* < 0.05.

**Figure 2** displays the interaction between the cue and flanking conditions. Follow-up analysis (adjusted *p* for eight comparisons <0.006) revealed significant RT differences between the congruent and incongruent conditions under all cue conditions, which confirms that ANT performance relies on executive control. As expected, no significant differences were observed between neutral and congruent flanking, with the exception of the spatial cue condition, in which the RT on the trials with a congruent cue was significantly longer than the RT on trials with a neutral cue.

Regarding error rate, a 4 × 3 ANOVA revealed a significant main effect for flanking condition [*F*(2,58) = 11.04, *p* < 0.003, partial η^2^ = 0.38]; the incongruent condition led to a higher error rate (5.30 ± 1.5%) than the neutral and congruent conditions, both of which showed no errors (0%). No main effect for cue (*F* = 1.84, *p* > 0.05) was observed, and no significant interaction was observed between cue and flanking condition (*F* = 2.78, *p* > 0.05).

#### Alerting Network

**FIGURE 3:**
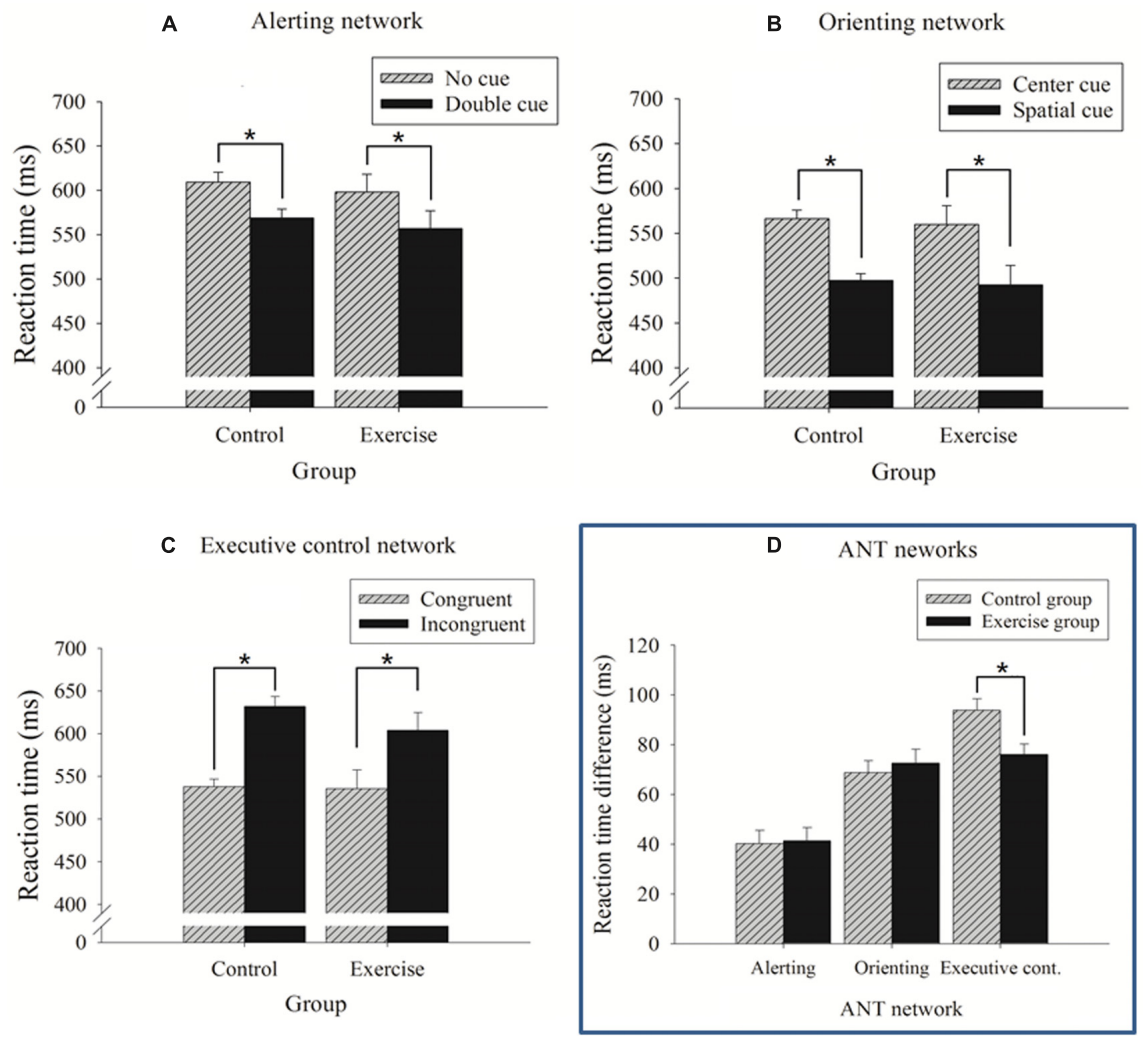
**Reaction time (RT) as function of the cue and flanking conditions for the control and exercise groups separately for each network (mean ± SE). (A)** Alerting network; **(B)** orienting network; and **(C)** executive control network. **(D)** RT differences between specific cue and flanking conditions that reflect the efficiency of the ANT networks for the control and exercise groups. ^∗^*p* < 0.05.

The 2 × 2 ANOVA revealed a significant main effect for cue (**Table 2**): the no cue condition exhibited a longer RT (603.94 ± 11.48 ms) compared with the double cue condition (563.12 ± 11.11 ms). No main effect of group or interaction with cue condition was observed (*p*s’ > 0.05; **Figure 3A**). No significant results were observed for errors rates (*p*s’ > 0.05).

**Table 2:** Summary of behavioral and neuroelectric results for the three attention networks.

Attention network	Dependent variable		Factor	ANOVA/*t*-test
Alerting	Behavioral	Reaction time (RT)	Cue condition (no/double)	*F*(1,28) = 119.20, *p* < 0.001, partial η^2^ = 0.83)
		Error rate	/	/
	Neuroelectric	P3 amplitude	Group (exercise/control)	*F*(1,28) = 6.46, *p* < 00.01, partial η^2^ = 0.24
			Cue condition (no/double)	*F*(1,28) = 12.34, *p* < 00.002, partial η^2^ = 0.38
			Electrode site (Fz/Cz/Pz)	*F*(2,56) = 8.25, *p* < .005, partial η^2^ = 0.29
			Cue × Electrode	*F*(2,56) = 37.59, *p* < 0.001, partial η^2^ = 0.65
		P3 latency	/	/
Orienting	Behavioral	RT	Cue condition (center/spatial)	*F*(1,28) = 373.00, *p* < 0.001, partial η^2^ = 0.94
		Error rate	/	
	Neuroelectric	P3 amplitude	Cue condition (center/spatial)	*F*(1,28) = 22.92, *p* < 0.001, partial η^2^ = 0.53
			Cue × Electrode	*F*(2,56) = 23.89, *p* < 0.001, partial η^2^ = 0.70
		P3 latency	Cue condition (center/spatial)	*F*(1,28) = 17.77, *p* < 0.001, partial η^2^ = 0.43
Executive control	Behavioral	RT	Flanking condition (congurent/incongruent)	*F*(1,28) = 751.95, *p* < 0.001, partial η^2^ = 0.97
			Group × Flanking	*F*(1,28) = 8.29, *p* < 0.008, partial η^2^ = 0.26
		Error rate	Flanking condition (congurent/incongruent)	*F*(1,28) = 15.59, *p* < 0.001
	Neuroelectric	P3 amplitude	Group (exercise/control)	*F*(1,28) = 5.62, *p* < 0.01, partial η^2^ = 0.22
		P3 latency	Flanking condition (congurent/incongruent)	*F*(1,28) = 7.76, *p* < 0.01, partial η^2^ = 0.24
			Flanking × Electrode	*F*(2,56) = 7.04, *p* < 0.004, partial η^2^ = 0.23
ANT network	Behavioral	RT difference	RTno cue – RT double cue	*t*(28) = 0.15, *p* > 0.05
			RTcenter cue - RTspatial cue	*t*(28) = 0.62, *p* > 0.05
			RTincongruent – RTcongruent	*t*(28) = -2.88, *p* < 0.008


Similarly, a *t-*test indicated no differences in the time values of the alerting network between the exercise and control groups (**Table 2**; **Figure 3D**).

#### Orienting Network

The 2 × 2 ANOVA revealed a significant main effect for cue (**Table 2**): the center cue condition exhibited a longer RT (560.71 ± 11.41 ms) than the spatial cue condition (490.01 ± 11.04 ms). No main effect for group or an interaction with cue condition was observed (*p*s*’* > 0.05; **Figure 3B**). No significant results were observed for error rates (*p*s’ > 0.05).

Similarly, a *t-*test indicated no differences in the time values of the orienting network between the exercise and control groups (**Table 2**; **Figure 3D**).

#### Executive Control Network

The 2 × 2 ANOVA revealed a significant main effect for flanking condition (**Table 2**): the incongruent condition exhibited a longer RT (619.04 ± 11.84 ms) than the congruent condition (534.02 ± 11.69 ms). The main effect for group did not reach significance (*p* > 0.05).

A significant interaction between the flanking condition and group was observed (**Table 2**): follow-up analysis of the interaction showed that both the exercise and control groups exhibited a significant congruency effect (*p*s’ < 0.001) but the magnitude or conflict effect was reduced in the exercise group (**Figure 3C**). The *t*-tests performed on the RT-differences between the congruent and incongruent trials consistently revealed that the exercise group had smaller conflict effects (76.10 ms) than the control group (93.95 ms), which reflects a higher efficiency of the executive control (**Table 2**; **Figure 3D**).

For error rates, a main effect for flanking condition was observed (**Table 2**), with higher error rates in the incongruent compared with congruent conditions (5.2 ± 0.1 versus 0.2 ± 0.00%). No significant Group × Flanking interaction was found.

### Neuroelectric Data

#### Alerting Network

Regarding the P3 peak amplitude, the 2 × 2 × 3 repeated-measure ANOVA revealed a main effect for group, in which the exercise group exhibited a larger P3 amplitude than the control group; a main effect for cue condition, in which the no cue condition exhibited a larger P3 amplitude than the double cue condition (10.16 ± 0.48 versus 8.09 ± 0.49 μV); a main effect for electrode site, in which Pz exhibited a larger P3 amplitude than Cz and Fz (10.31 ± 0.59 versus 9.54 ± 0.47 and 7.54 ± 0.61 μV); and a significant interaction between cue condition and electrode site (**Table 2**). No other significant interaction effects were observed. The follow-up analysis of the Cue × Electrode interaction revealed that under the no cue condition, the P3 amplitude was larger compared with the double cue condition at Cz and Pz but not at Fz. No other effects were observed.

Regarding the P3 latency, the three-way ANOVA did not reveal significant effects (*p* > 0.05).

#### Orienting Network

Regarding the P3 amplitude, the three-way mixed-model ANOVA revealed a main effect for cue, in which the center cue condition exhibited a larger P3 amplitude than the spatial cue condition (11.28 ± 0.61 versus 7.88 ± 0.62 μV), and a significant interaction between cue condition and electrode site (**Table 2**). No main effect for group or electrode site or other significant interaction effects were observed. The follow-up analysis of the Cue × Electrode interaction revealed that under the center cue condition, the P3 amplitude was larger compared with the spatial cue condition at Cz and Pz but not at Fz.

Regarding the P3 latency, the three-way ANOVA revealed a main effect for cue condition (**Table 2**); the center cue condition exhibited a shorter P3 latency than the spatial cue condition (346.83 ± 13.66 versus 414.56 ± 12.99 ms). Neither a main effect for group, nor other main effects or significant interactions were observed (*ps* > 0.05).

#### Executive Control Network

**FIGURE 4:**
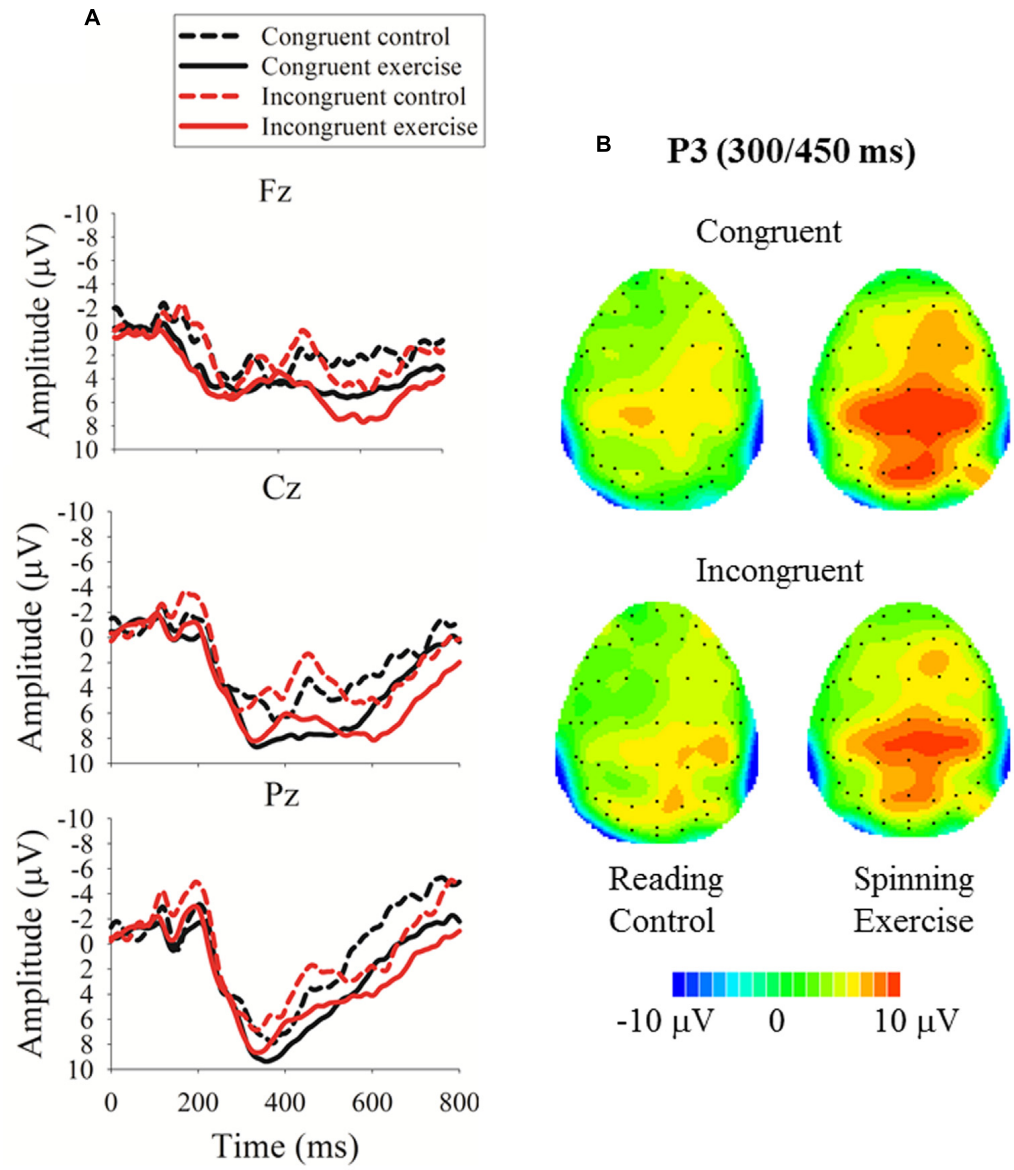
**Event-related brain potential (ERP) data for the executive network task. (A)** Grand averaged ERPs at Fz, Cz, and Pz for the exercise and control groups; **(B)** topographic maps of the voltage differences in the P3 component (300–500 ms) between the exercise and control groups.

**FIGURE 5:**
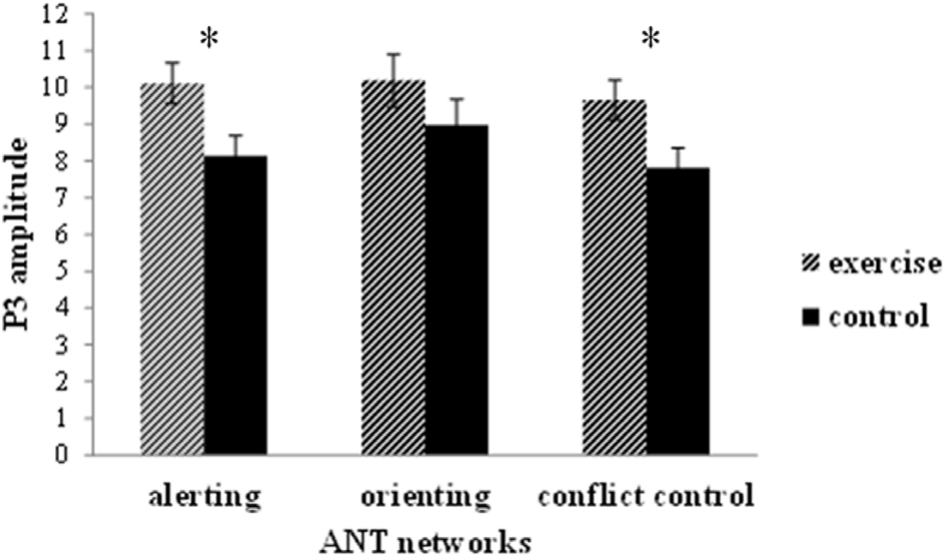
**Peak amplitude (mean and SE) of the P3 component of stimulus-locked ERPs for the alerting, orienting, and executive control networks in the ANT**. Data are collapsed across midline electrodes (Fz, Cz, Pz) and presented separately for the exercise and the control group. ^∗^*p* < 0.05.

Regarding the P3 amplitude, the three-way mixed-model ANOVA revealed a main effect for group (**Table 2**): the exercise group exhibited a larger P3 amplitude than the control group (**Figures 4 and 5**). No other main effects or significant interactions (*p* > 0.05) were observed.

Regarding the P3 latency, the three-way ANOVA revealed a main effect for flanking condition, in which the congruent condition exhibited a shorter P3 latency than the incongruent condition (371.28 ± 13.48 versus 403.10 ± 17.35 ms), and a significant interaction between flanking condition and electrode site (**Table 2**). No other main or interaction effects were observed. The follow-up analysis of the Flanking × Electrode interaction revealed that under the incongruent condition, the P3 latency was longer compared to the congruent condition at Fz. No other effects were observed.

## Discussion

The present study aimed to investigate whether and how an acute bout of moderate intensity spinning exercise influences the efficiency of the three attention networks that perform the functions of alerting, orienting and executive control. To understand the specific component attention processes and underlying mechanisms that are transiently altered by spinning exercise participation, the multifunctional ANT was employed, and neuroelectric and behavioral data were jointly considered. On the whole, The results suggest that after spinning, more resources are allocated to task-relevant stimuli, which translates into better attention performance. However, although neuroelectric indices exhibited enhanced resource allocation after spinning for both the alerting and executive control networks, this finding was paralleled by a performance improvement only in the executive control network.

### Acute Exercise and Executive Control Network

These differentiated neuroelectric and behavioral effects of acute exercise on the executive control network are intriguing. Neuroelectrically, acute exercise probably enhanced arousal and ‘freed’ processing resources, as indicated by a larger P3, distributed across congruent and incongruent task conditions (**Figure 5**), which is consistent with previous ERP studies of acute exercise effects on executive control (Hillman et al., 2003; Kamijo et al., 2004, 2007). Behaviorally, however, exercise benefited attention performance in only the incongruent condition, as indicated by the fact that the individuals who had just exercised had a lower conflict effect. It appears that after acute exercise, brain resources were evenly rendered available across more and less executive control demanding conditions. Nevertheless, behavioral benefits were not observed when the task was less challenging, most likely because of a floor effect, but the benefits emerged when increased attentional resources could help counteract conflict effects. This selective response speed improvement after exercise on incongruent trials is not attributable to a shift in the speed-accuracy trade-off setpoint because it was not paralleled by an increment in response errors.

The enhanced efficiency of the executive control network that emerged from the present study suggests that the increased availability of neural resources might be a mediator of the acute exercise effects on executive control. Moreover, the fact that an improvement in performance was selectively observed only in the executively challenging conflict condition suggests that cognitive task complexity may be a moderator of whether the brain resources that are rendered available immediately after acute exercise may be optimally exploited to enhance attentional performance (Pesce, 2009). It is also important to consider that the participants were amateur athletes who regularly practiced basketball, which is a strategic sport that requires cognitive expertise. Sport-related cognitive expertise does not appear to generally amplify acute exercise benefits on attention but does increase the selectivity of exercise-related benefits. For example, soccer players (Pesce et al., 2007b) experienced the beneficial effects of exercise on attentional performance only under more difficult task conditions that required a higher degree of executive control. Based on these and similar results, Pesce (2009) proposed that how the increased amount of resources ‘freed’ by an acute bout of exercise are allocated to the ongoing attentional task depends on the interplay of the cognitive task complexity and individual cognitive expertise.

However, Pesce et al.’s (2007a,b, 2011) studies were in-task exercise research. Regarding the executive control network, there is a clear behavioral and neuroelectrical divergence between the results of in-task (Pontifex and Hillman, 2007) and off-task exercise research (Hillman et al., 2003; Kamijo et al., 2004, 2007). In-task studies have indicated either no modulation of the conflict effect by exercise (Davranche et al., 2009) or impaired conflict resolution under exercise that was attributed to the need of resource sharing between the cognitive task and whole-body movement control under dual-task conditions (Pontifex and Hillman, 2007). Thus, negative dual task effects may have outweighed any attentional benefit derived from acute exercise consistently observed in off-task research (Hillman et al., 2003; Kamijo et al., 2004, 2007).

While the P3 amplitude modulation by exercise was consistent with the findings of ERP studies of acute exercise effects on conflict control (Hillman et al., 2003; Kamijo et al., 2004, 2007), the absence of an exercise effect on the P3 latency was inconsistent. This finding may have occurred because in the present study, the conflict control was studied within the ANT, which integrates measures of alerting, orienting, and conflict control. Recent neuroelectric evidence (Galvao-Carmona et al., 2014) calls for caution when using ANT to perform an estimation of executive control functioning, because it integrates non-comparable experimental conditions relying on the interaction of multiple attention networks. However, it is beyond the scope of this study to investigate the interaction between the attention networks with respect to acute exercise. Because flanker-congruency effects may vary under different alerting and orienting conditions (Fan et al., 2009), the evidence obtained with tasks that tap executive control alone is not fully comparable.

### Acute Exercise, Alerting, and Orienting Networks

A further neural consequence of the spinning exercise bout was a larger P3 amplitude in the alerting network task (**Figure 5**). Because exercise is a generally arousing activity (Cahill and Alkire, 2003), it is not surprising that the functioning of the alerting network was modulated by the intensity and duration of our spinning exercise workload. However, this finding was not paralleled by a performance advantage. The absence of an acute exercise effect on the behavioral efficiency of the alerting network, but the presence of a performance benefit after exercise for the executive control network is at odds with the results of the Huertas et al. (2011) RT study (17), which demonstrated a beneficial effect of acute exercise on alerting but not on executive control performance.

However, several methodological differences do not enable thorough comparisons. Huertas et al. (2011) used a modified ANT with auditory stimuli to tap alerting effects and employed in-task exercise. Similar to previous evidence (Smit et al., 2005), Huertas et al. (2011) demonstrated that moderate aerobic exercise benefited RT performance more pronouncedly in no-tone compared with tone trials, which led to a reduction of the alerting effect (Huertas et al., 2011). They argued that due to the physiological state of readiness (tonic vigilance) and consequently enhanced reaction speed by physical effort, the phasic alertness effect of the tone was smaller (i.e., floor effect in RT). Moreover, participants in Huertas et al.’s (2011) study were highly skilled cyclist who performed a cycling task; this variable may have modulated their arousal level, intimately linked to alerting functioning. The basketball players’ ANT performance may have been differently influenced by spinning that is a non-specific exercise type with respect to the players’ usual sport practice. The divergence of results reinforces the view that different mechanisms act on the relationship between attention and an antecedent or a concomitant exercise bout (Chang et al., 2012).

Divergences among the present and other studies also emerged as concerns the orienting network. This may be due to methodological differences that do not allow to draw overall conclusions on acute exercise effects on the orienting network. Such differences regard not only the time relation between physical exercise and cognitive testing, but also the time elapsing between cue and target onset SOA, the informative value of the spatial cue and the participants’ individual fitness level. The only two studies in which, similar to the present, exercise after-effects on spatial orienting were tested in fit young adults (Sanabria et al., 2011; Llorens et al., 2014), an exercise-induced modulation of orienting was found. However, in those studies, orienting effects were elicited exogenously by means of non-informative cues at different SOAs, whereas in the present study, the spatial cue location always validly predicted where the stimulus was to appear and generated a RT facilitation that seemed not influenced by acute spinning exercise.

### Conclusion, Implications, and Future Directions

The present study is novel in terms of both methods and outcomes that contribute to the following issues needing further research: (1) the nature of the physical and cognitive task performances and (2) the time at which the cognitive task is administered relative to the acute exercise bout. To the best of our knowledge, this is the first attempt to study the after-effects of acute exercise on the multiple attention networks via a multifunctional attentional task. Joining behavioral and neuroelectric outcomes has shown consistencies and inconsistencies that provide information regarding the potential mediators and moderators of the effects of acute moderate exercise on attention performance. The main finding suggests that rather than eliciting a general improvement, a single bout of spinning exercise selectively serves to enhance executive control of attention through a beneficial influence to stimulus engagement aspects.

Notably, our ERP results revealed an exercise-related modulation of neuroelectric resources allocated to task-relevant stimuli (i.e., P3 amplitude), regardless of cue/flanker conditions and scalp sites, for both the alerting and executive control networks. This neural correlate of acute exercise after-effects was similar to what found in previous studies with younger and older adults (Hillman et al., 2003; Kamijo et al., 2009) and even with typically or atypically developing children (Pontifex et al., 2013). Our neuroelectric data extend those findings by presenting a higher resolution of scalp distribution (i.e., 64 channels) that complements what we have specifically found as an increment of the amount of allocated neuroelectric resources reflected in the P3 amplitude modulation. Since, however, only three midline sites have been used for ERP computation, future studies with a higher number of selected channels (e.g., over the prefrontal cortex of both hemispheres) is warranted to further our understanding of acute exercise effects on brain activity and higher-level cognition.

The study also has limitations that need to be addressed. First, the relatively low sample size that might have prevented detecting exercise effects on alerting and orienting. Also, the participants’ individual characteristics (fitness and sport skill level) and quantitative physical exercise characteristics (intensity and duration) were not manipulated, but specifically selected to yield the largest effects from an exercise bout. Also, the spinning exercise effects were not compared with the effects of a different physical exercise modality.

Furthermore regarding neuroelectric data, only the amplitude and latency parameters of the P3 waveform of the ERPs were analyzed. Although this ERP component is the most frequently examined in the context of exercise and cognition research (Hillman et al., 2009), further components that have been used to evaluate the relationship between chronic exercise and attention (Pesce and Bosel, 2001; Taddei et al., 2012) should also be considered in acute exercise research. Moreover, ANT studies have also benefited from the use of event-related functional magnetic resonance imaging to explore the brain areas involved in the three attention systems targeted by the ANT (Fan et al., 2005), as well as brain oscillations and power spectrum analyses, because each network has a distinct oscillatory activity and time course (Fan et al., 2007). Therefore, further studies that explore acute exercise effects on attentional networks with these methodologies are warranted.

The spinning exercise protocol had unconventional/uncontrolled elements due to the choice to trade control for ecological validity. Given the relatively large amount of highly controlled cycling exercise studies, we considered beneficial to present a novel protocol that has a higher ecological validity. To find an optimal control-reality trade-off setpoint, in further studies, the workload might be individualized based on a spinning-specific estimation of cardiovascular fitness performance. Future research should also employ variable intensity spinning protocols, closer to real spinning exercise training, with manipulation of the magnitude of the intensity fluctuations and with order reversal (Kang et al., 2007) and cognitive measurements during recovery at different time points (Hung et al., 2013). Also, related to this last point, not only the time relation between exercise cessation and beginning of cognitive testing, but also potential effects of ‘time on task’ during cognitive testing should be taken into account ^1^.

The present findings, along with future applied research, will certainly contribute to the knowledge regarding the effects of commonly practiced physical training bouts, such as spinning, on specific aspects of attention that are relevant for daily living activities. For instance in work settings, active breaks of aerobic spinning exercise performed before executive attention-demanding tasks may be an advantage from an ergonomic perspective, thus contributing to the issue of physical and mental health and efficiency at workplace.

## Author Contributions

Y-KC: (1) substantial contribution to the conception and design of the work and to data interpretation; (2) drafting Statistical analysis and Results and revising Introduction and Discussion sections critically; (3) final approval of the version to be published; (4) agreement to be accountable for all aspects of the work.

CP: (1) substantial contribution to data interpretation; (2) drafting Introduction and Discussion and revising Statistical analysis and Results sections critically; (3) final approval of the version to be published; (4) agreement to be accountable for all aspects of the work.

Y-TC: (1) substantial contribution to data acquisition and analysis; (2) drafting Statistical analysis and Results sections; (3) final approval of the version to be published; (4) agreement to be accountable for all aspects of the work.

C-YK: (1) substantial contribution to data acquisition and analysis; (2) drafting Statistical analysis and Results sections; (3) final approval of the version to be published; (4) agreement to be accountable for all aspects of the work.

D-YF: (1) substantial contribution to the conception and design of the work and to data interpretation; (2) drafting Statistical analysis and Results and revising Introduction and Discussion sections critically; (3) final approval of the version to be published; (4) agreement to be accountable for all aspects of the work.

## Conflict of Interest Statement

The authors declare that the research was conducted in the absence of any commercial or financial relationships that could be construed as a potential conflict of interest.

## Footnotes

1 We thank the reviewers for their proactive thinking to identify outlooks for future research.

## References

[B1] American College of Sports Medicine. (2013). *ACSM’s Guidelines for Exercise Testing and Prescription*, 9th Edn. New York: Lippincott Williams and Wilkins. 10.1249/JSR.0b013e31829a68cf 23851406

[B2] Bauman A. Bull F. Chey T. Craig C. L. Ainsworth B. E. Sallis J. F. (2009). The international prevalence study on physical activity: results from 20 countries. *Int. J. Behav. Nutr. Phys. Act.* 6 21 10.1186/1479-5868-6-21 PMC2674408 19335883

[B3] Cahill L. Alkire M. T. (2003). Epinephrine enhancement of human memory consolidation: interaction with arousal at encoding. *Neurobiol. Learn. Mem.* 79 194–198 10.1016/S1074-7427(02)00036-9 12591227

[B4] Callejas A. Lupiáñez J. Tudela P. (2004). The three attentional networks: on their independence and interactions. *Brain Cogn*. 54 225–227 10.1016/j.bandc.2004.02.012 15050779

[B5] Cereatti L. Casella R. Manganelli M. Pesce C. (2009). Visual attention in adolescents: facilitating effects of sport expertise and acute physical exercise. *Psychol. Sport Exerc.* 10 136–145 10.1016/j.psychsport.2008.05.002

[B6] Chang Y. K. Chi L. Etnier J. L. Wang C. C. Chu C. H. Zhou C. L. (2014). Effect of acute aerobic exercise on cognitive performance: role of cardiovascular fitness Psychol. *Sport Exerc.* 15 464–470 10.1016/j.physbeh.2011.06.005

[B7] Chang Y. K. Labban J. D. Gapin J. I. Etnier J. L. (2012). The effects of acute exercise on cognitive performance: a meta-analysis. *Brain Res.* 1453 87–101 10.1016/j.brainres.2012.02.068 22480735

[B8] Davranche K. Audiffren M. (2004). Facilitating effects of exercise on information processing. *J. Sports Sci.* 22 419–428 10.1080/02640410410001675289 15160595

[B9] Davranche K. Hall B. McMorris T. (2009). Effect of acute exercise on cognitive control required during an Eriksen flanker task. *J. Sport Exerc. Psychol.* 31 628–639. 20016112 10.1123/jsep.31.5.628

[B10] Etnier J. L. Chang Y. K. (2009). The effect of physical activity on executive function: a brief commentary on definitions, measurement issues, and the current state of the literature. *J. Sport Exerc. Psychol.* 31 469–483. 19842543 10.1123/jsep.31.4.469

[B11] Fan J. Byrne J. Worden M. S. Guise K. G. McCandliss B. D. Fossella J. (2007). The relation of brain oscillations to attentional networks. *J. Neurosci.* 27 6197–6206 10.1523/jneurosci.1833-07.2007. 17553991 PMC6672149

[B12] Fan J. Gu X. Guise K. G. Liu X. Fossella J. Wang H. (2009). Testing the behavioral interaction and integration of attentional networks. *Brain Cogn.* 70 209–220 10.1016/j.bandc.2009.02.002 19269079 PMC2674119

[B13] Fan J. McCandliss B. D. Fossella J. Flombaum J. I. Posner M. I. (2005). The activation of attentional networks. *Neuroimage* 26 471–479 10.1016/j.neuroimage.2005.02.004 15907304

[B14] Fan J. McCandliss B. D. Sommer T. Raz A. Posner M. I. (2002). Testing the efficiency and independence of attentional networks. *J. Cogn. Neurosci.* 14 340–347 10.1162/089892902317361886 11970796

[B15] Galvao-Carmona A. González-Rosa J. J. Hidalgo-Muñoz A. R. Páramo D. Benítez M. L. Izquierdo G. (2014). Disentangling the attention network test: behavioral, event related potentials, and neural source analyses. *Front. Hum. Neurosci.* 8:813 10.3389/fnhum.2014.00813 PMC4195286 25352800

[B16] Hillman C. H. Kamijo K. Scudder M. (2011). A review of chronic and acute physical activity participation on neuroelectric measures of brain health and cognition during childhood. *Prev. Med*. 52(Suppl. 1), S21–S28 10.1016/j.ypmed.2011.01.024 PMC3094734 21281669

[B17] Hillman C. H. Pontifex M. Themanson J. R. (2009). “Acute aerobic exercise effects on event-related brain potentials,” in *Exercise and Cognitive Function,* eds McMorris T. Tomporowski P. D. Audiffren M. (West Sussex: John Wiley and Sons), 161–178.

[B18] Hillman C. H. Snook E. M. Jerome G. J. (2003). Acute cardiovascular exercise and executive control function. *Int. J. Psychophysiol.* 48 307–314 10.1016/S0167-8760(03)00080-1 12798990

[B19] Huertas F. Zahonero J. Sanabria D. Lupianez J. (2011). Functioning of the attentional networks at rest vs. during acute bouts of aerobic exercise. *J. Sport Exerc. Psychol.* 33 649–665. 21984640 10.1123/jsep.33.5.649

[B20] Hung T. M. Tsai C. L. Chen F. T. Wang C. C. Chang Y. K. (2013). The immediate and sustained effects of acute exercise on planning aspect of executive function. *Psychol. Sport Exerc.* 14 728–736 10.1016/j.psychsport.2013.05.004

[B21] Hüttermann S. Memmert D. (2014). Does the inverted-U function disappear in expert athletes? An analysis of the attentional behavior under physical exercise of athletes and non-athletes. *Physiol. Behav.* 131 87–92 10.1016/j.physbeh.2014.04.020 24747278

[B22] Kamijo K. Hayashi Y. Sakai T. Yahiro T. Tanaka K. Nishihira Y. (2009). Acute effects of aerobic exercise on cognitive function in older adults. *J. Gerontol. Ser. B Psychol. Sci. Soc. Sci.* 64 356–363. 19363089 10.1093/geronb/gbp030

[B23] Kamijo K. Nishihira Y. Hatta A. Kaneda T. Wasaka T. Kida T. (2004). Differential influences of exercise intensity on information processing in the central nervous system. *Eur. J. Appl. Physiol.* 92 305–311 10.1007/s00421-004-1097-2 15083372

[B24] Kamijo K. Nishihira Y. Higashiura T. Kuroiwa K. (2007). The interactive effect of exercise intensity and task difficulty on human cognitive processing. *Int. J. Psychophysiol.* 65 114–121 10.1016/j.ijpsycho.2007.04.001 17482699

[B25] Kang J. Mangine G. T. Ratamess N. A. Faigenbaum A. D. Hoffman J. R. (2007). Influence of intensity fluctuation on exercise metabolism. *Eur. J. Appl. Physiol.* 100 253–260 10.1007/s00421-007-0424-9 17323070

[B26] Lambourne K. Tomporowski P. (2010). The effect of exercise-induced arousal on cognitive task performance: a meta-regression analysis. *Brain Res.* 1341 12–24 10.1016/j.brainres.2010.03.091 20381468

[B27] Llorens F. Sanabria D. Huertas F. (2014). The influence of acute intense exercise on exogenous spatial attention depends on physical fitness level. *Exp. Psychol.* 30 1–10. 10.1027/1618-3169/a000270 25270559

[B28] Lopez-Minarro P. A. Muyor Rodríguez J. M. (2010). Heart rate and overall ratings of perceived exertion during spinning cycle indoor session in novice adults. *Sci. Sports* 25 238–244 10.1016/j.scispo.2009.11.003

[B29] Mahoney C. R. Hirsch E. Hasselquist L. Lesher L. L. Lieberman H. R. (2007). The effects of movement and physical exertion on soldier vigilance. *Aviat. Space. Environ. Med*. 78 B51–B57. 17547304

[B30] McMorris T. (2008). Exercise and cognition: towards an inter-disciplinary model. *Open. Sports Med. J.* 2 60–68 10.2174/1874387000802010060

[B31] Memmert D. (2009). Pay attention! A review of visual attentional expertise in sport. *Int. Rev. Sport Exerc. Psychol.* 2 119–138 10.1080/17509840802641372

[B32] Neuhaus A. H. Urbanek C. Opgen-Rhein C. Hahn E. Ta T. M. Koehler S. (2010). Event-related potentials associated with Attention Network Test. *Int. J. Psychophysiol.* 76 72–79 10.1016/j.ijpsycho.2010.02.005 20184924

[B33] Pesce C. (2009). “An integrated approach to the effect of acute and chronic exercise on cognition: the linked role of individual and task constraints,” in *Exercise and Cognitive Function*, eds Mcmorris T. Tomporowski P. D. Audiffren M. (West Sussex: John Wiley and Sons), 213–226.

[B34] Pesce C. Bosel R. (2001). Focusing of visuospatial attention: electrophysiological evidence from subjects with and without attentional expertise. *J. Psychophysiol.* 15 256–274 10.1027//0269-8803.15.4.256

[B35] Pesce C. Cereatti L. Casella R. Baldari C. Capranica L. (2007a). Preservation of visual attention in older expert orienteers at rest and under physical effort. *J. Sport Exerc. Psychol.* 29 78–99. 17556777 10.1123/jsep.29.1.78

[B36] Pesce C. Tessitore A. Casella R. Pirritano M. Capranica L. (2007b). Focusing of visual attention at rest and during physical exercise in soccer players. *J. Sports Sci.* 25 1259–1270 10.1080/02640410601040085 17654238

[B37] Pesce C. Cereatti L. Forte R. Crova C. Casella R. (2011). Acute and chronic exercise effects on attentional control in older road cyclists. *Gerontology* 57 121–128 10.1159/000314685 20453491

[B38] Pontifex M. B. Hillman C. H. (2007). Neuroelectric and behavioral indices of interference control during acute cycling. *Clin. Neurophysiol.* 118 570–580 10.1016/j.clinph.2006.09.029 17095295

[B39] Pontifex M. B. Hillman C. H. (2008). Neuroelectric measurement of cognition during aerobic exercise. *Methods* 45 271–278 10.1016/j.ymeth.2008.04.003 18762137

[B40] Pontifex M. B. Saliba B. J. Raine L. B. Picchietti D. L. Hillman C. H. (2013). Exercise improves behavioral, neurocognitive, and scholastic performance in children with attention-deficit/hyperactivity disorder. *J. Pediatr.* 62 543–551 10.1016/j.jpeds.2012.08.036 23084704 PMC3556380

[B41] Posner M. I. Petersen S. E. (1990). The attention system of the human brain. *Annu. Rev. Neurosci.* 13 25–42 10.1146/annurev.ne.13.030190.000325 2183676

[B42] Posner M. I. Rothbart M. K. (2007). Research on attention networks as a model for the integration of psychological science. *Annu. Rev. Neurosci.* 58 1–23. 10.1146/annurev.psych.58.110405.085516 17029565

[B43] Sanabria D. Morales E. Luque A. Gálvez G. Huertas F. Lupiañez J. (2011). Effects of acute aerobic exercise on exogenous spatial attention. *Psychol. Sport Exerc.* 12 570–574 10.1016/j.psychsport.2011.04.002

[B44] Smit A. S. Eling P. A. Hopman M. T. Coenen A. M. (2005). Mental and physical effort affect vigilance differently. *Int. J. Psychophysiol*. 57 211–217 10.1016/j.ijpsycho.2005.02.001 16109291

[B45] Taddei F. Bultrini A. Spinelli D. Di Russo F. (2012). Neural correlates of attentional and executive processing in middle-age fencers. *Med. Sci. Sports Exerc.* 44 1057–1066 10.1249/MSS.0b013e31824529c2 22157879

